# Evaluation of the automatic ELISA Triturus analyser

**DOI:** 10.1155/S1463924603000051

**Published:** 2003

**Authors:** S. Blanco, J. Domínguez, O. Jiménez, D. Sánchez, N. Galí, L. Matas, V. Ausina, R. Galimany

**Affiliations:** 1 Servicio de Microbiología Hospital Universitari Germans Trias i Pujol Facultad de Medicina de la Universidad Autónoma de Barcelona Ctra. Canyet s/n, Badalona Barcelona E-08916 Spain; 2 Servicio de Bioquímica Hospital Universitari Germans Trias i Pujol Facultad de Medicina de la Universidad Autónoma de Barcelona Barcelona Spain


**Journal of Automated Methods & Management in Chemistry**
**Vol. 25, No. 2 (March–April 2003) pp. 31–34**

Taylor & Francis

Taylor & Francis Group

### Evaluation of the automatic ELISA Triturus analyser

**S. Blanco[1], J. Domínguez[1], O. Jiménez[2], D. Sánchez[1], N. Galí[1], L. Matas[1]*, V. Ausina[1] and R. Galimany[2]**

[1]*Servicio de Microbiología i* [2]*Bioquímica, Hospital Universitari Germans Trias i Pujol, Facultad de Medicina de la Universidad Autónoma de Barcelona, Barcelona, Spain*

*The objective was to evaluate the automatic ELISA Triturus analyser in order to assess its practicability and imprecision. The analyser was evaluated according to the guidelines of the Spanish Society of Clinical Chemistry. The evaluation was performed in two steps: evaluation of the imprecision and the inaccuracy of a semiquantitative and qualitative technique, and study of the practicability. The within-run imprecision rate ranged from 8.9 (VC) to 10.2% (VC) for the semiquantitative test, and from 11 (VC) to 17.2% (VC) for the qualitative one. The between-run imprecision rate ranged from 6 (VC) to 9.7% (VC) for the semiquantitative test, and from 8.8 (VC) to 18% (VC) for the qualitative technique. No sample-related carryover was detected in negative samples. The relative inaccuracy was correct for both techniques. Non-specific binding caused by waste products from the analysed circuits was not detected. The Triturus analyser is a reliable open analytical system with a considerable working capability, rendering this apparatus adequate for conventional laboratories with a medium-to-high work charge.*

### Introduction

The Triturus Analyser (Diagnostics Grifols, S.A., Barcelona, Spain) is an immunoassay system. The evaluation reported here was performed according to the guidelines of the Spanish Society of Clinical Biochemistry and Molecular Pathology (SEQC) [1–5]. It included the study of within- and between-run imprecision, sample-related carryover, method comparison with patients' specimens, detection limit, and non-specific binding. The analyser's practicability was also evaluated.

### Experimental

### *Instrumentation description*

The Triturus Analyser is an open, fully automated, multi-test and -batch immunoassay system. The instrument can perform all steps of a microplate assay including: sample dilution, reagent addition, washings, absorbance reading and results calculation. Programming at every step is also possible.

Several tests can be performed simultaneously on a given sample and the system presents the capability of independently scheduling several consecutive groups of tests. The system can be interfaced with the user's in-house computer for data management and reporting.

### *Statistical analysis*

Data were analysed using the program SPSS 8.0 for Windows.

### *Reagents*

The reagents used in this evaluation were as follows:

- *Toxoplasma* IgG test set (SeraQuest™, North Miami, FL 33181, USA). It is an enzyme-linked immunosorbent assay (ELISA) for the semiquantitative detection of human immunoglobulin (Ig) G antibodies to *Toxoplasma gondii*. The test includes one positive and negative control and two calibrators. The index value or International Units (IU) $ml^{-1}$ of each sample was calculated by extrapolation in a calibration curve. The calibration curve is performed using two calibrators included by the manufacturer in the kit and using one blank. The interpretation of the results was the following: $< 27$ IU $ml^{-1}$ as negative, between 27 and 33 IU $ml^{-1}$ as equivocal, and $> 33$ as positive.
- Captia™ Epstein–Barr viral Capsid Antigen (EBV VCA P-18) IgG (Trinity Biotech, Jamestown, NY, USA). This test is an ELISA for the qualitative determination of IgG antibodies in human serum to VCA antigen. The technique includes a negative control, two positive controls (high and low control) and three cut-off calibrators used to calculate the test sample index: Test sample index = test sample absorbance/(mean absorbance cut-off calibrator × correction factor). The correction factor was determined by the manufacturer for each lot in order to account for day-to-day fluctuations in assay activity due to room temperature and timing. The interpretation of the results was as follows: $< 0.90$ IU $ml^{-1}$ as negative, between 0.90 and 1.09 IU $ml^{-1}$ as equivocal, and $> 1.09$ as positive.

### *Controls and samples*

Human sera containing 0.1% sodium azida were used as controls to validate the performance of the test at three distinct levels. They were supplied by Quest International, Inc. (North Miami, FL 33181, USA). To assess the method comparison, clinical serum specimens were tested.

*To whom correspondence should be addressed. Lourdes Matas, Servicio de Microbiología, Hospital Universitario 'Germans Trias i Pujol', Ctra. Canyet s/n, E-08916 Badalona, Barcelona, Spain. e-mail: lmatas@ns.hugtip.scs.es

*Journal of Automated Methods & Management in Chemistry* ISSN 1463–9246 print/ISSN 1464–5068 online © 2003 Taylor & Francis Ltd
http://www.tandf.co.uk/journals
DOI: 10.1080/1463924031000066221

*Evaluation protocol*

*Imprecision.* To evaluate the within-run imprecision, three different samples of control sera were tested. Fifteen replicates of each control sera were tested in three different runs performed on consecutive days, while the between-run imprecision was studied by using three different samples of control sera distributed in 20 different runs performed on consecutive days.

*Inaccuracy*

*Sample-related carryover.* The carryover caused by the sample was studied following an alternative permutation order between five control samples with different concentration. Two high concentration specimens (A) followed by three low concentration specimens (B) were processed 10 times (A1 A2 B1 B2 B3).

*Method comparison.* To assess the method comparison, 107 and 95 serum specimens were tested by EBV IgG ELISA (Gull Laboratories, Inc., Salt Lake City, UT, USA) and VIDAS TOXO IgG (BioMérieux S.A., Marcy-l'Etoile, France), respectively, as reference methods. The samples ranged within the analytical interval of both tests. The statistical evaluation was undertaken by a non-parametric method according to Passing and Bablock [6, 7].

The VIDAS Toxo IgG is an automated assay for the VIDAS system that enables anti-*Toxoplasma* IgG in human serum to be quantitatively measured. The assay principle combines the enzyme immunoassay method with a final fluorescent detection. The test includes one positive control, one negative control and one calibrator. The results are analysed automatically and the anti-*Toxoplasma* IgG sample concentration is calculated by the VIDAS system. The results are expressed in IU $ml^{-1}$ in relation to a calibration curve stored in the VIDAS system memory and are interpreted following the manufacturer's instructions.

The EBV IgG ELISA test is intended for the quantitative and qualitative detection of IgG antibody to the sp 125 viral capsid antigen of Epstein–Barr virus in human serum by the ELISA method. The test includes negative and positive controls, three calibrators and a reference serum. The test was performed using the manual procedure. Results were interpreted following the manufacturer's recommendations.

*Detection limit.* The detection limit study was only applied in quantitative techniques. It was calculated as 3 SDs of a blank concentration obtained from 20 determinations of 20 analytical series.

*Non-specific binding.* To correct the non-specificity of the antibodies used in the techniques, distilled water was used as samples instead of the specimens.

### Results and discussion

*Imprecision*

Tables 1 and 2 show the results of the within- and between-run imprecision studies obtained from the *Toxoplasma* and Epstein–Barr tests, respectively. Results from both tests were acceptable for this kind of techniques. The within-run imprecision rate ranged from 8.9 (VC) to 10.2% (VC) for the semiquantitative test, and from 11 (VC) to 17.2% (VC) for the qualitative assay. The between-run imprecision rate ranged from 6 (VC) to 9.7% (VC) for the semiquantitative test, and from 8.8 (VC) to 18% (VC) for the qualitative method.

*Inaccuracy*

*Sample-related carryover.* No contamination effect was detected in negative samples caused by carryover from positive samples in either of the techniques. The means of B1 and B3 were calculated and compared using the Wilcoxon test [2]. The resulting standard variations were not significant ($p > 0.05$).

*Method comparison.* The quantitative method (*Toxoplasma* IgG test) presented a good correlation with the reference method to a level of 150 IU $ml^{-1}$ (figure 1). From this point, which corresponds to absorbance units near 2.5, the optical reader of the analyser looses linearity; results higher than this value were non-comparable.

Sensitivity and specificity of the quantitative technique (table 3) and the qualitative one (table 4) were determined using the reference methods by means of a cross-tab. The qualitative test (Captia™ Epstein–Barr viral Capsid Antigen) correlated with the reference method (table 4).

*Table 1. Variation coefficient (VC) results of within- and between-run imprecision from the* Toxoplasma *test (SeraQuest™) performed by the automatic ELISA Triturus Analyser. The within- and between-run studies were developed using three different levels of concentrations of control sera.*

| Level | Within-run VC (%) | Between-run VC (%) |
|---|---|---|
| 1 | 9.3 | 6 |
| 2 | 10.2 | 9.7 |
| 3 | 8.9 | 6.7 |

*Table 2. Variation coefficient (VC) results of within- and between-run imprecision from the Epstein–Barr test (Trinity Biotech) performed by the automatic ELISA Triturus Analyser. The within- and between-run studies were developed using three different levels of concentrations of control sera.*

| Level | Within-run VC (%) | Between-run VC (%) |
|---|---|---|
| 1 | 11 | 8.8 |
| 2 | 17 | 18 |
| 3 | 17.2 | 10.7 |

| | Coeficient | 95% Confidence interval |
|---|---|---|
| **Intersection** | 37,582 | 25,310 to 52,193 |
| **Slope** | 1,025 | 0,808 to 1,284 |
| **p** | < 0.01 | |

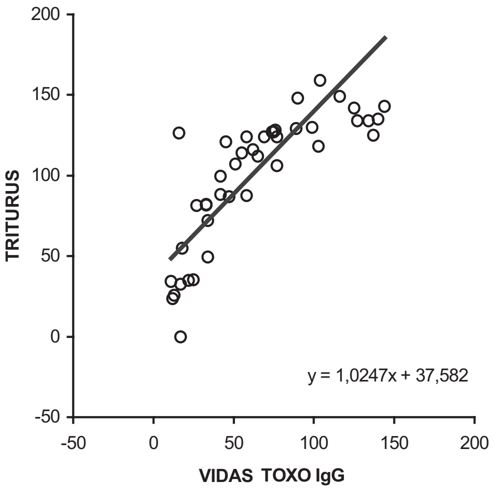

*Figure 1. Correlation results of the quantitative test (*Toxoplasma *IgG, SeraQuest$^{TM}$) by using Triturus with the reference method VIDAS TOXO IgG test by VIDAS.*

*Table 3. Sensitivity, specificity and predictive value of the* Toxoplasma IgG test *(SeraQuest$^{TM}$) by comparing the results with the reference method by means of a crosstab.*

| | Vidas Toxo IgG | | |
|---|---|---|---|
| Triturus | Positive | Negative | Total |
| Positive | 52 | 0 | 52 |
| Negative | 3 | 40 | 43 |
| Total | 55 | 40 | 95 |
| Sensitivity | 94.5% | | |
| Specificity | 100.0% | | |
| Positive predictive value | 100.0% | | |
| Negative predictive value | 93.0% | | |
| Efficiency | 96.8% | | |

*Table 4. Sensitivity, specificity and predictive value of the Epstein–Barr IgG test (Trinity Biotech) by comparing the results with the reference method by means of a crosstab.*

| | Gull EBV Ig G ELISA | | |
|---|---|---|---|
| Triturus | Positive | Negative | Total |
| Positive | 55 | 6 | 61 |
| Negative | 5 | 41 | 46 |
| Total | 60 | 47 | 107 |
| Sensitivity | 91.0% | | |
| Specificity | 87.0% | | |
| Positive predictive value | 90.0% | | |
| Negative predictive value | 89.0% | | |
| Efficiency | 89.0% | | |

*Detection limit.* The detection limit of the quantitative technique was 0.125 IU $ml^{-1}$ with a mean of 0.053 IU $ml^{-1}$ and a SD of 0.023.

*Non-specific binding.*

Non-specific bindings caused by waste products from the analysed circuits were not detected.

*Practicability*

*Environmental factors.* The Triturus Analyser is large and requires quite a large room for installation. It produces noise at a low level and operates at room temperature. An uninterruptible power supply is needed to prevent the analyser from stopping when a fluctuation of alternating current occurs.

*Operators' training.* Technique programming and unexpected problem resolution requires a good training period and experience. The instrument is easily and quickly understood.

User's information systems:

- Easy to use access screen.
- Screen shows which step the analyser is performing.
- Clear error messages appear on the screen.

*Starting delay of the system.* The staff begins the daily start-up procedure, which takes about 5 min.

It is recommended that a water system washing is performed before starting and at the end of each working day, although the Triturus does an automatic priming before of each series.

*Calibration mode.* This depends on the ELISA technique, since each assay includes its own calibrators.

*Specimens' loading.* A 24-h availability is needed to process urgent samples intercalated in a previously programmed specimen series. Eight different techniques can be performed on one sample, provided all the protocols are compatible. The specimen has to be processed in tubes with a maximum diameter of 12–13 mm and 75 mm height. Primary tubes can be used if presenting these characteristics. It can use a barcode for direct sample identification.

*Processing speed.* It depends on the EIA test. In our study, a maximum of 92 specimens could be processed in 2–3 h.

*Methods.* This analyser provides only one measure system: spectrophotometry at 405, 450, 492, 550, 600 and 620 nm wavelengths. The measure methods available are the following: cut-off, end-point, curve interpolation (point by point, lineal regression, polynomic regression, spline, 4PL, lin–log and log–log.)

*Information processing.* Simple data input. Possible direct communication with the laboratory central computer.

Screen results edition. Allows their correction and validation. Results can be filed by series and by sample, identification is by the laboratory number. The presentation of the results is by sample and by technique. In addition, it is possible to consultant the calibration curve and the blank and controls results.

*Versatility and flexibility*. This is an open analytical system that allows working with different EIA simultaneously provided they are compatible. It is necessary to program the steps of each test. Its use does not require any special facilities. Conventional laboratories can use this analyser. There is the possibility of choosing disposable or fixed tips. Blanks, calibrators, positive and negative controls can be included.

### Discussion

The practicability assessment presents the analyser as an open analytical system with the possibility of multiserial performance; it allows working with different EIA tests simultaneously and has a considerable working capability.

The need for the calibration of each technique and series in open systems and ELISA techniques represents a work increase and requires experience. The analyser allows one to perform multiparametric series, provided the requested analytical parameters are compatible.

The analyser presents correct reliable results, being the quantitative technique results better than those obtained by the qualitative test. This is due to the quality of the reagents of the qualitative assay. According to the imprecision and accuracy results of the quantitative method, the use of the automatic analyser does not affect reliability. The reliable results point out that the analyser is accurate and precise, with within- and between-run imprecision acceptable for this type of apparatus.

According to the practicability and reliable results, the Triturus Analyser is adequate for conventional laboratories with medium-to-high work charge. The analyser has the capability to apply multiparametric series provided compatible protocols are used.

### Acknowlegement

The authors thank Diagnostic Grifols, S.A. for the provision of all the materials needed for the evaluation.

### References

1. Spanish Society of Clinical Chemistry, *Química Clínica*, **12**, (1993) 511.
2. Alumà, A., Alsina, M. J., Armenter, C., Bertrán, N., Biosca, C., Doladé, M., Farré, J., Galimany, R., Paz, J. M., Taberner, L. and Martínez, M., in *Selección y evaluación de sistemas analíticos*, 6 (Barcelona: Spanish Society of Clinical Chemistry, 1996) 45.
3. Biosca, C. and Galimany, R., in *Selección y evaluación de sistemas analíticos*, 7 (Barcelona: Spanish Society of Clinical Chemistry, 1995), 57.
4. Spanish Society of Clinical Chemistry, *Química Clínica*, **17** (1998), 247.
5. Spanish Society of Clinical Chemistry, *Química Clínica*, **17** (1998), 258.
6. Passing, H. and Bablock, W., *J. Clin. Chem. Clin. Biochem.*, **21** (1983), 709.
7. Passing, H. and Bablock, W., *J. Clin. Chem. Clin. Biochem.*, **22** (1984), 431.

